# DNA Phosphorothioate Modifications Are Widely Distributed in the Human Microbiome

**DOI:** 10.3390/biom10081175

**Published:** 2020-08-12

**Authors:** Yihua Sun, Lingxin Kong, Guojun Wu, Bo Cao, Xiaoyan Pang, Zixin Deng, Peter C. Dedon, Chenhong Zhang, Delin You

**Affiliations:** 1 State Key Laboratory of Microbial Metabolism, Joint International Research Laboratory of Metabolic and Developmental Sciences, and School of Life Sciences & Biotechnology, Shanghai Jiao Tong University, Shanghai 200240, China; 13162732720@163.com (Y.S.); konglingxin7@163.com (L.K.); wgj@sjtu.edu.cn (G.W.); caobogxu@126.com (B.C.); xypang@sjtu.edu.cn (X.P.); zxdeng@sjtu.edu.cn (Z.D.); 2 Department of Biological Engineering and Center for Environmental Health Science, Massachusetts Institute of Technology, Cambridge, MA 02139, USA; pcdedon@mit.edu; 3 Antimicrobrial Resistance Interdisciplinary Research Group, Singapore-MIT Alliance for Research and Technology, 1 CREATE Way, Singapore 138602, Singapore

**Keywords:** DNA modification, DNA phosphorothioation, microbiome, gut microbiome

## Abstract

The DNA phosphorothioate (PT) modification existing in many prokaryotes, including bacterial pathogens and commensals, confers multiple characteristics, including restricting gene transfer, influencing the global transcriptional response, and reducing fitness during exposure to chemical mediators of inflammation. While PT-containing bacteria have been investigated in a variety of environments, they have not been studied in the human microbiome. Here, we investigated the distribution of PT-harboring strains and verified their existence in the human microbiome. We found over 2000 PT gene-containing strains distributed in different body sites, especially in the gastrointestinal tract. PT-modifying genes are preferentially distributed within several genera, including *Pseudomonas*, *Clostridioides*, and *Escherichia*, with phylogenic diversities. We also assessed the PT modification patterns and found six new PT-linked dinucleotides (C_ps_G, C_ps_T, A_ps_G, T_ps_G, G_ps_C, A_ps_T) in human fecal DNA. To further investigate the PT in the human gut microbiome, we analyzed the abundance of PT-modifying genes and quantified the PT-linked dinucleotides in the fecal DNA. These results confirmed that human microbiome is a rich reservoir for PT-containing microbes and contains a wide variety of PT modification patterns.

## 1. Introduction

Phosphorothioate (PT) DNA modifications, in which the nonbridging oxygen in the phosphate backbone is replaced by sulfur, are widespread among bacteria and archaea [1]. The PT-modifying gene cluster, *dndA-E*, has been found in more than 1300 sequenced genomes [2] and confers cells with 5′-G_ps_AAC-3′/5′-G_ps_TTC-3′ or 5′-G_ps_GCC-3′ consensus sequences [3]. Recently, another PT-modifying gene cluster has been reported, *sspA-D*, which confers cells with 5′-C_ps_CA-3′ on the single strand [4]. The DndA protein, which can be functionally substituted by an IscS (a cysteine desulfurase located elsewhere in the genome) [5,6], transfers sulfur into the Fe-S cluster of DndC [5,7]. SspA and SspD may share the same initial sulfur mobilization pathway with DndA and DndC, respectively [4]. Meanwhile, SspC shows ATPase activity and may provide energy in a manner similar to DndD [4]. Though PT modifications are usually introduced in a sequence-specific manner, only 12%–14% of target motifs are modified [3,8]. In some bacteria, PT modifications are involved in a restriction-modification (R-M) system with gene cassette *dndF-H/sspE* or an antiviral system with gene cassette *pbeA-D* [9]. However, nearly half of the PT-containing strains lack *dndF-H/sspE* and *pbeA-D*, indicating that the PT modifications possess other functions [4,10]. A recent transcriptomic and metabolomic analysis showed that PT modifications contribute to the cellular redox state [11], while, at the same time, PT modifications confer sensitivity to the HOCl produced by neutrophils [12]. To date, little is known about the presence of PT-containing bacteria in the human microbiome. Here, we performed an informatic analysis of sequenced microbiome-related genomes and a mass spectrometric analysis of fecal DNA to show the widespread presence of PT-modifying genes and PT-linked dinucleotides in the human microbiome.

## 2. Materials and Methods

### 2.1. Multiple Sequence Alignments

All publicly available genomes (including the complete and partial genomes) were downloaded from the online databases of NCBI (National Center for Biotechnology Information) (https://www.ncbi.nlm.nih.gov/genome/), EMBL (European Molecular Biology Laboratory) (https://www.ebi.ac.uk/), and HMP (Human Microbiome Project) (https://www.hmpdacc.org/). We used the MakeDB of multigeneblast (software version 1.1.13) [13] to convert the original databases into the GBK format. The *dnd* genes of *Salmonella enterica serovar* Cerro 87 (CP008925: 3477655...3481641) and the *ssp* genes of *Vibrio cyclitrophicus* FF75 (NZ_ATLT01000001: 2194844...2200061) were used as PT-modifying component queries [4]. The *DndF-H* cluster of *S. enterica serovar* Cerro 87 (CP008925: 3467758…3475796) was used as the restriction component query. The *PbeA-D* cluster of *Haloterrigena jeotgali* A29 (CP031303: 460843…466000) was used as the antiviral component query. The amino acid translation of each gene sequence within the query gene cluster is searched against the selected GBK (Genebank) database, yielding a dataset of BLAST (Basic Local Alignment Search Tool) hits [13]. The BLAST hits are then mapped to their parent nucleotide scaffolds, based on the information from the database [13]. The nucleotide scaffolds are then sorted according to their empirical similarity scores with the query gene cluster [13]. The number of blast hits per gene to be mapped is 250. The weight of synteny conversion in hit sorting is 0.5. The minimal sequence coverage of blast hits is 25. The minimal identity of blast hits is 30%. The maximum distance between genes in locus is 20 kb. These parameters above were set up for multiple sequence alignments in multigeneblast (software version 1.1.13) [13].

### 2.2. Phylogenic Analysis

The identification of protein sequences was based on the TIGR (The Institute for Genomic Research) database (http://www.tigr.org) annotation (e.g., TIGR03233, TIGR03183, TIGR03185, and TIGR03184 for DndA, B, C, D, and E, respectively) [14]. The amino acid sequences of DndC/SspD (accession numbers were shown in dataset) were downloaded from the online databases (e.g., https://www.ncbi.nlm.nih.gov/genome/). Then. we combined these amino acid sequences with the DndC/SspD of *V. cyclitrophicus* FF75 (C_ps_C) and *Pseudomonas fluorescens* Pf0-1 (G_ps_G) and aligned them by MEGA7 with the maximum likelihood method (500 bootstrap replications). The phylogenic tree was visualized using iTOL (http://itol.embl.de/).

### 2.3. Gene Abundance Calculation

A thenon-redundant human gut microbial gene catalog was constructed in our previous study [15]. In brief, the reads from the metagenomes were de novo assembled into contigs. Genes were predicted from the contigs and merged into non-redundant genes based on sequence similarity. The abundance of the genes was obtained by mapping the reads on the catalog, obtaining the gene-length normalized base counts, and adjusting the sequencing depth with a resampling procedure. All the genes were clustered into CAGs based on their abundance data using the canopy-based algorithm with default parameters. CAGs with more than 700 genes were regarded as bacterial CAGs for further analysis. The CAG abundance profiles were calculated as the sample-wise median gene abundance, essentially as described elsewhere [16].

### 2.4. Fecal DNA Preparation

All subjects gave their informed consent for inclusion before they participated in the study [15]. The study was conducted in accordance with the Declaration of Helsinki, and the protocol was approved by the Ethics Committee of the School of Life Sciences and Biotechnology, Shanghai Jiao Tong University (No. 2012-016). Feces samples were obtained and immediately frozen on dry ice upon collection and stored at −80 °C until further analysis [15]. DNA extraction from the human fecal samples was conducted as previously described [15] and purified by the QIAamp DNA mini kit (51304, QIAGEN, Germany).

### 2.5. Detection of PT-Linked Dinucleotides

All the fecal DNA samples were hydrolyzed with 4 units of nuclease P1 (Sigma, St. Louis, MO, USA) and subsequently dephosphorylated by 10 units of alkaline phosphatase (Fermentas), essentially as described elsewhere [17]. For qualitation, the digested DNA samples were pre-purified by reversed-phase high-performance liquid chromatography on a ThermoHypersil Gold Aq column (250 × 4.6; SN:1292941W) at a flow rate of 0.8 mL/min with the following parameters: solvent A: water with 8 mM NH_4_OAC; B: Acetonitrile; gradient: 3% B for 17 min; 40% B for 23 min; 100% B for 10 min; 3% B for 10 min. The pre-purified samples were dried and re-suspended in 50 μL of deionized water for analysis by the Agilent 6410 Triple Quad liquid chromatography mass spectrometer, as previously described [18].

For quantification, the digested DNA samples were purified by ultrafiltration, dried, and resuspended in 40 μL of deionized water. Then, the mixture containing PT-dinucleotides was resolved on an Agilent ZORBAX SB-C18 column (2.1 × 150 mm, 3.5 μm bead size) with a flow rate of 0.3 mL/min and the following parameters: column temperature: 25 °C; solvent A: 0.1% formic acid in H_2_O; solvent B: 0.1% formic acid in acetonitrile; gradient: 4% B for 5 min, 4% to 15% B over 15 min, 15 to 20% B for 5 min, and 20 to 100% B for 5 min. The high-performance liquid chromatography column was coupled to an Agilent G6470A Triple Quadrupole mass spectrometer with an electrospray ionization source in positive mode with the following parameters: gas flow, 10 L/min; nebulizer pressure, 30 psi; drying gas temperature, 325 °C; and capillary voltage, 3000 V. Multiple reaction monitoring modes were used for the detection of ions derived from the precursor ions, with all the instrument parameters optimized for maximal sensitivity (retention time in min, precursor ion *m*/*z*, fagmentor voltage, product ion *m*/*z* and collision energy for qualitation, product ion *m*/*z* and collision energy for quantification): d(A_ps_A), 10. 4, 581, 102 V, 348, 18 V, 136. 1, 38 V; d(A_ps_C), 11.3, 557, 102 V, 81.1, 42 V, 136, 30 V; d(A_ps_G), 11. 9, 597, 118 V, 81.1, 54 V, 152, 22 V;d(A_ps_T), 13.1, 572, 102 V, 81.1, 50 V, 136, 18 V; d(C_ps_A), 9. 5, 557, 102 V, 348.1, 18 V, 136, 30 V; d(C_ps_C), 8.5, 533, 86 V, 81.1, 42 V, 112 V, 22 V; d(C_ps_G), 9.3, 573, 118 V, 81.1, 74 V, 152.1, 30 V; d(C_ps_T), 12.8, 548, 86 V, 81, 54 V, 112, 14 V; d(G_ps_A), 15.8, 597.14, 118 V, 81.1, 12 V, 136, 32 V; d(G_ps_C), 11.9, 573, 86 V, 152.1, 26 V, 112, 34 V; d(G_ps_T), 18.2, 588.13, 102 V, 81.1, 24 V, 152, 75V; d(G_ps_G), 12.4, 613, 102 V, 462.1, 8 V, 152.1, 30 V;d(T_ps_A), 18. 0, 572, 118 V, 81.1, 74 V, 136, 18 V; d(T_ps_C), 16. 0, 548, 102 V, 81.1, 66 V, 112, 18 V; d(T_ps_G), 13. 0, 588, 70 V, 135, 70 V, 152.1, 18 V; d(T_ps_T), 15.2, 563, 110 V, 81.1, 56 V, 127, 37 V. The hydrolyzed mono nucleosides were quantified by high-performance liquid chromatography, as described [8].

## 3. Results

### 3.1. PT Genes Are Widely Distributed in the Human Microbiome

**Figure 1:**
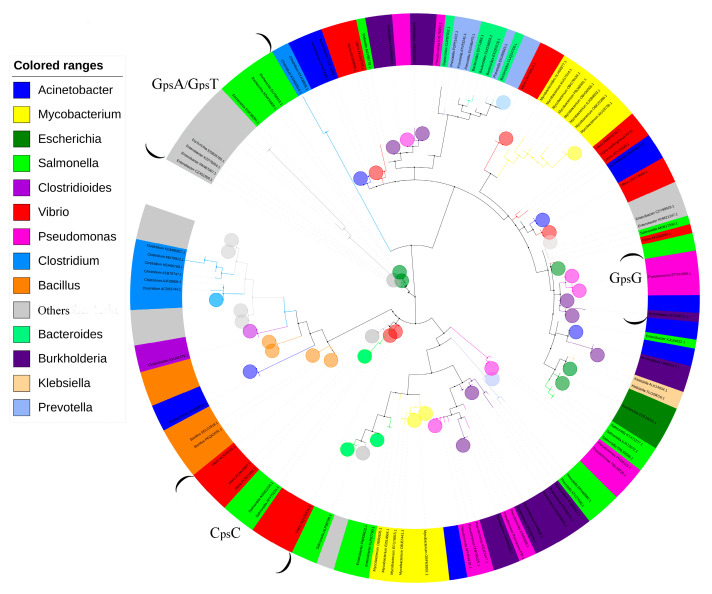
Phylogenetic distribution of the strains containing PT-modifying genes. The DndC/SspD in 2623 strains were created by MEGA7 with the maximum likelihood method with 500 bootstrap replications and was visualized using iTOL. *V. cyclitrophicus* FF75 contains C_ps_C, and *P. fluorescens* Pf0-1 contains G_ps_G. *E. coli* B7A and *S. enterica serovar* Cerro 87 both contain G_ps_A/G_ps_T.

We searched for PT-modifying genes in over 40,000 bacterial genomes under the human microbiome category from websites (https://www.ncbi.nlm.nih.gov/genome/, https://www.ebi.ac.uk/, and https://www.hmpdacc.org/). Multiple sequence alignments were performed using the genomes for strains harboring PT-modifying genes. A total of 2621 strains harboring PT-modifying genes (dataset) were obtained from the results queried by the PT-modifying genes. The amino acid sequences of DndC/SspD were used to construct a phylogenetic tree (Figure 1). The strains in the same genus are clustered into different branches according to sequence similarity (Figure 1). The bubbles represent collapsed clades and each bubble clustered strains with sequence identities over 60%. These bubbles were used to show the distribution of homogeneous sequences due to the strong correlation between modifying types and amino acid sequences [17]. The PT-linked dinucleotides of G_ps_A/G_ps_T, G_ps_G, and C_ps_C were separated on relatively independent clades with sequence identities over 30%. The boundaries of these clades were defined by the brackets (Figure 1). It was reported that strains with the same PT-linked dinucleotides were presumed to be closer on the phylogenetic tree [11]. Intriguingly, over 60% of the strains were clustered on distinct branches far from the clades marked with PT-linked dinucleotides (Figure 1). Thus, we speculated that unknown modification patterns could exist in the human microbiome.

We speculated that PT-harboring species spread across the entire human body due to the distribution of PT genes in diverse species (Figure 1). Thus, we collected bacterial genomes directly from distinct anatomical sites on the human body (https://www.hmpdacc.org/) to perform multiple sequence alignments. Although the total number of genomes is limited, we found diverse species harboring PT in four body parts (Table 1). Notably, there are species differences in the distribution of *dnd* genes and *ssp* genes (Table 1). Intriguingly, the PT-modifying gene-harboring strains in the genus *Prevotella* were found only in the oral and urogenital systems (Table 1), despite the relatively abundant distribution of *Prevotella* species in the human intestinal tract [19]. All the data above (Figure 1, Table 1) confirm that strains harboring PT-modifying genes are widely distributed in the human body.

### 3.2. Species Difference of PT-Related Genes

**Figure 2:**
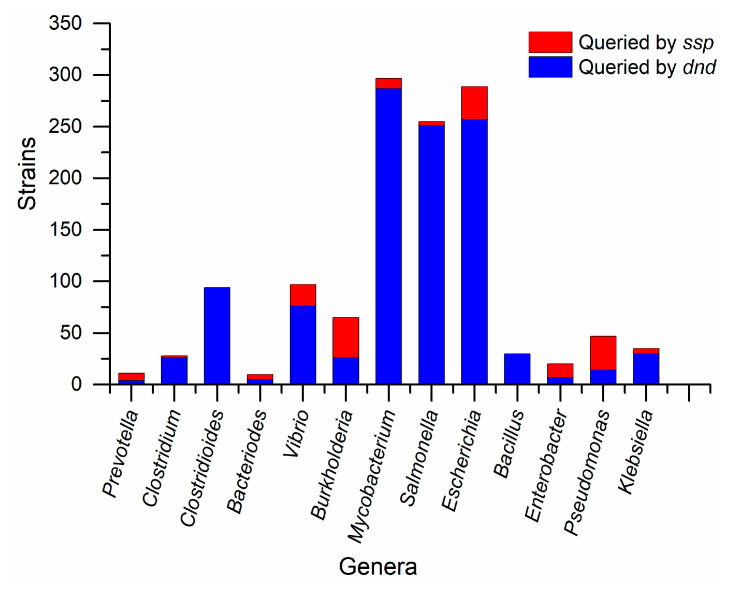
Distribution of PT-modifying gene cluster in different genera. Query genes were from *S. enterica serovar* Cerro 87 (CP008925: 3477655...3481641) and *V. cyclitrophicus* FF75 (NZ_ATLT01000001: 2194844...2200061), respectively.

**Figure 3:**
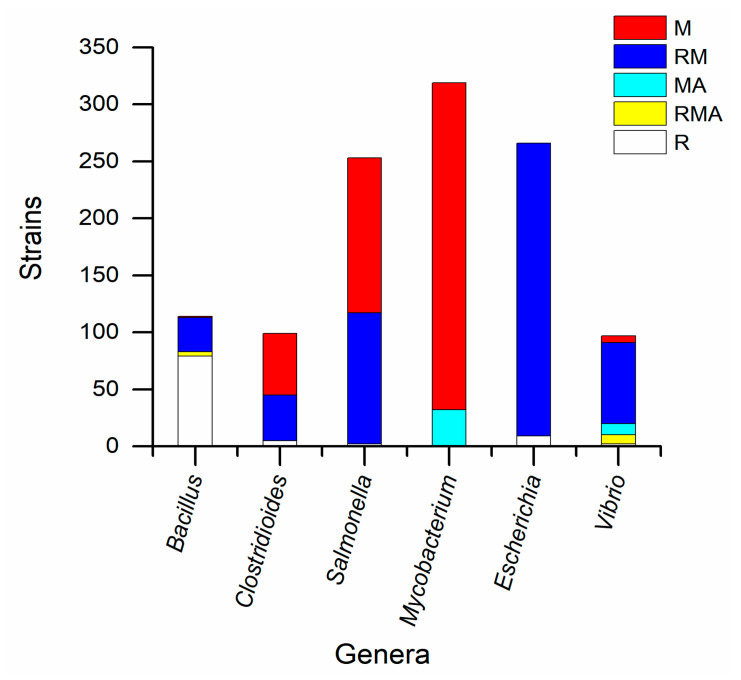
Distribution of PT-related gene cassettes in different genera. “R” means strains harboring *dndF-H* without PT-modifying genes. “M” means strains harboring PT-modifying genes without *dndF-H*. “RM” means strains harboring *dndF-H* and PT-modifying genes. “MA” means strains harboring PT-modifying genes and *pbeA-D*. “RMA” means strains harboring PT-modifying genes, *dndF-H* and *pbeA-D*.

It has been reported that PT gene clusters can be spread by horizontal transfer [17,20], and we find that the distribution of PT-modifying genes varied among genera. For instance, over 90% of the *Mycobacterium* strains harbor *dnd* genes rather than *ssp* genes (Figure 2). On the other hand, over 60% of the *Pseudomonas* strains harbor *ssp* genes rather than *dnd* genes (Figure 2). Notably, *dnd* genes are widely distributed in *Mycobacterium*, *Salmonella,* and *Escherichia* rather than the other genera (Figure 2). To further investigate the role of PT, we performed a multiple sequence alignment for *dndF-H* and *PbeA-D.* Notably, the *dndF-H* gene cassette was “missing” in 90% of strains of *Mycobacterium,* which means that most *Mycobacterium* strains lack the R component (Figure 3). On the contrary, the *dndF-H* gene cassette was widely distributed in *Escherichia* strains. Meanwhile, the *pbeA-D* gene cassette existed in 10% of the *Mycobacterium* and *Vibrio*, which suggests that most human microorganisms lack the antiviral component (Figure 3). We speculated that PT may play other roles in these species without both *dndF-H* and *PbeA-D*. It seems that there exist undiscovered PT-dependent genes in their genomes. On the contrary, over 60% of the *Bacillus* strains harbor the R component without any modifying genes (Figure 3), which means these strains harbor undiscovered modifying systems to protect their DNA against nickase [3].

### 3.3. Atypical PT Gene Clusters of Different Genera

**Figure 4:**
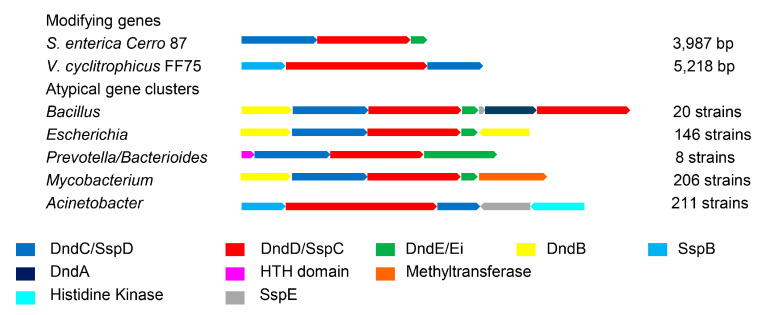
Atypical PT gene clusters in different genera. Genes are indicated as arrows and are colored based on their predicted function. DndC/SspD: ATP pyrophosphatase. DndD/SspC: ATPase. DndE/Ei: helicase. DndB: regulatory protein. SspB: nickase. DndA: cysteine desulfurase. SspE: nickase.

The organization of the PT-related genes varied among the genera (Figure 4). In 146 strains of *E. coli* and 20 strains of *Bacillus cereus*, the genes surrounding the PT-modifying genes encoded homologs of *dndA* (cysteine desulfurase) [5], *dndB* (negative transcriptional regulator) [18], and *dndD* (possibly related to DNA structure alteration) [21,22]. For instance, the identities are 23.98% between DndB and its homolog from *E. coli* Ecol_316 (NCBI: CP018957). Genomic islands may facilitate the horizontal transfer of PT systems and result in multiple copies of genes for *E*. *coli* and *B*. *cereus* [23]. Several strains from *Bacteroidales* (*Prevotella* and *Bacteroides*) showed a rare *dndEi* in PT-modifying genes that have an additional DNA helicase domain compared to canonical DndE [24]. In 211 *Acinetobacter* strains, the histidine kinase genes are adjacent to the SspE, which is the R component of the *ssp* system [4]. We speculate that the histidine kinase genes (Figure 4) involve in a two-component system to regulate the expression of SspE. Moreover, it was previously reported that there are complex interactions between DNA methylation and PT [25]. Intriguingly, 72% of PT-modifying genes in *Mycobacterium* strains lack both *dndF-H* and *pbeA-D* (Figure 3) and are adjacent to methyltransferase genes (Figure 4). This suggests that there is a shared mechanism linking the R system between DNA methylation and the PT [25] in *Mycobacterium* strains. These results indicate that PT plays various roles in different species.

### 3.4. Detection of PT-Linked Dinucleotides in Human Fecal DNA

For DNA methylation, restriction-modification systems are classified into four types depending on multiple factors, including subunits and target motifs [26]. In bacteria, the sequence specificity of DNA methylation depends on the target recognition domains (TRDs) of the modifying complex, and the variation in the TRDs impacts the sequence specificity upon modification [27]. For PT, although the TRDs have not been demonstrated, there is a strong correlation between the PT sequence contexts and modifying complex sequences [17]. Based on the variation in the *dndC*/*sspD* genes (Figure S1) and the phylogenic distribution of PT-linked dinucleotides (Figure 1), it remains a possibility that there are undiscovered modifying patterns, including different motifs and PT-linked dinucleotides. Exploiting the fact that most strains possessing PT-modifying genes were assigned to the gut microbiome (Table 1), we initiated a search for new PT consensus sequences by analyzing the diversity of PT-linked dinucleotides in human fecal DNA. Notably, six new PT-linked dinucleotides (C_ps_G, C_ps_T, A_ps_G, T_ps_G, G_ps_C, A_ps_T) were detected from human fecal DNA (Table 2, Figure S2). Additionally, two PT nucleotides (C_ps_A, T_ps_A) previously discovered in mutants were detected, which were previously discovered in *E. coli* DH10B expressing PT-modifying genes from *S. enterica* 87 [17].

### 3.5. The Abundance of PT Modifications Varied Among Individuals

**Figure 5:**
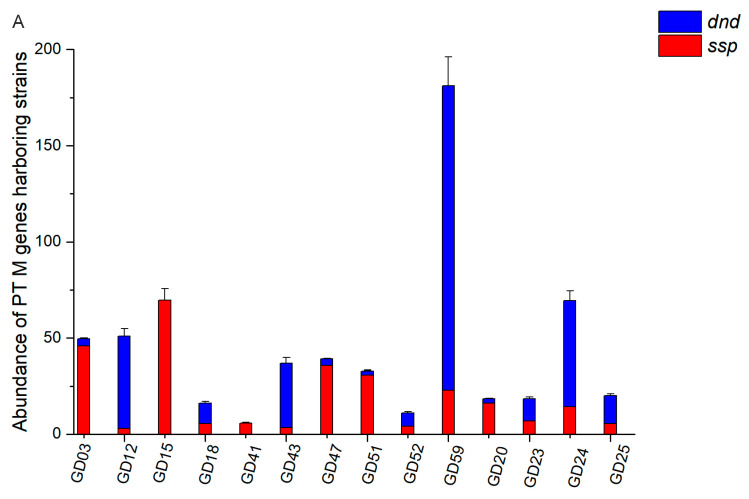

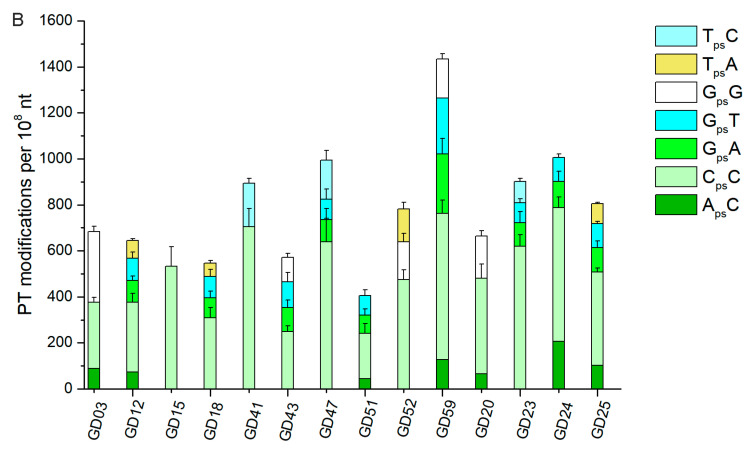
(**A**)The abundance of strains harboring PT-modifying genes in the fecal DNA from 14 individuals. (**B**) Quantification of PT-linked dinucleotides in fecal DNA from 14 individuals. Data are shown as mean ± SD. “dnd” means strains harboring *dnd* genes. “ssp” means strains harboring *ssp* genes.

Although we could detect PT-linked dinucleotides in the fecal DNA, the abundance of PT genes and the modification types remain unknown in the gut microbiome without quantitative analysis. Meanwhile, we speculated that the distribution of the *dnd*/*ssp* genes and modification patterns varied among individuals. Thus, we performed a quantitative analysis of the PT genes and modifications in human fecal DNA. Based on the metagenomic sequencing performed on 109 human fecal DNA samples, a gene catalog with ~ 2 million non-redundant human gut microbial genes was constructed [15]. By BLAST alignment, 209 potential PT genes with at least 30% identity and 80% coverage compared with the PT references were identified. We were able to assemble 118 high-quality draft genomes of prevalent gut bacteria from these metagenomic data. Among these bacterial genomes, nine harbored PT genes: *Bacteroides plebeius* CAG00296, *Bacteroidesplebeius* CAG0079, *Klebsiella sp.* CAG00146, and *Faecalibacterium prausnitzii* CAG00158 were queried by *dnd* genes, while *Bacteroides sp.* CAG00020, *Eubacterium ventriosu* CAG00166, *Clostridium bolteae* CAG00012, Clostridiales bacterium CAG00239, and *Clostridiales bacterium* CAG00048 were queried by *ssp* genes. To further investigate the PT landscapes in the human gut microbiome, we analyzed the strains harboring PT-modifying genes (dataset) in the 14 individuals (Figure 5A). Then, we quantified the PT-linked dinucleotides in these fecal samples and found seven different PT-dinucleotides (Figure 5B).

## 4. Discussion

Over 80% of the strains harboring PT-modifying genes could be assigned to Actinobacteria, Proteobacteria, and Bacteroidetes [11]. Notably, our survey in the human microbiome shows that PT-modifying genes are distributed in these phyla (Figure 1). The phylogenic tree (Figure 1) was used to demonstrate the distribution of homogeneous sequences rather than the genera distribution, though it exhibits the horizontal transfer of PT-modifying genes, which is consistent with the previous conclusions [28]. Although we could not demonstrate DndC/SspD-containing TRDs, the DndC/SspD may play the role of site recognition according to their function of inserting sulfur into the sugar-phosphate backbone of DNA [4]. Thus, we built the tree by DndC/SspD and assumed that the human microbiome contained new PT-linked dinucleotides due to the phylogenic distribution of G_ps_A/G_ps_T, G_ps_G, and G_ps_C (Figure 1). Fortunately, we discovered new PTs in human fecal DNA. We speculated that 5 μg of fecal DNA was not enough to detect multiple PT-linked dinucleotides. Thus, we tested 30 μg of fecal DNA with a control group of 1493. However, we found no more new PT-linked dinucleotides compared to 5 μg of fecal DNA. Additionally, the variety of PT-linked dinucleotides varied among the fecal DNA samples, which suggested that the PT-containing strains carried by each person could be classified through metadata. As shown in Table 2, z1 and z3 both carried G_ps_T, assigned to species queried by *dnd* genes (Figure 1); z2 and z3 both carried C_ps_C and A_ps_C, assigned to species queried by *ssp* genes (Figure 1); z2 and z3 both carried A_ps_T, assigned to unknown species. The qualitation and quantification of PT-linked dinucleotides confirmed the existence of PT in the human gut microbiome with a strong correlation between the PT-modifying gene abundance and the modified dinucleotide yield. Thus, the abundance of PT modifications varied among individuals due to the different quantities of PT-harboring bacteria.

Intriguingly, our data showed that the PT-modifying genes seemed widely distributed in different pathogens or opportunistic pathogens, such as the strains that belonged to *A. baumannii*, *P. aeruginosa, C. difficile*, and *M. abscessus* (Dataset). The different bioinformatic approaches including this work confirmed that PT-modifying genes widely spread in human microbiome-associated strains, especially pathogenic candidates [11,20,23]. The previous study reported that nearly half of the US clinical isolates of *M. abscessus* exhibited the PT phenotype during pulsed field gel electrophoresis [29]. It was also reported that PT-modifying genes were frequently found in pathogenic *E. coli* [28]. One of the possible causes is that PT modifications were presumed to enhance the antioxidative ability of these bacteria against environmental stress [14,30]. In contrast, halogens could induce PT-linked DNA breaks, which implicated that PT reduced the fitness for bacterial pathogens during human infections [12]. Thus, it remains a possibility that the microenvironment or drugs may intervene in the abundance of bacteria with PT in the human microbiome, while there are few related reports. Additionally, there are few reports about how PT-modifying genes spread in human pathogens or opportunistic pathogens.

## 5. Conclusions

PT confers bacteria with multiple physiological functions, making them a specific microbial population sharing unusual characteristics [10,11,12]. However, our understanding of PT-containing bacteria tends to be restricted to isolated strains rather than a community in the ecosystem. This study, to our knowledge, is the first time to systematically investigate the wide distribution of PT-containing microbes in the human microbiome. Our results firstly reveal the landscape of PT modifications in the gut microbiome. This work will guide future research on the transmission of PT among microbial communities in the micro-ecosystem of the human body. With the rapid growth of the human microbiome database, more PT-containing bacteria could be found to analyze the routine of PT transmission and discover the unknown motive force behind this phenomenon with effects on human health.

## Figures and Tables

**Table 1:** Strains containing PT-modifying genes from the human body.

Body Parts	Queried by *dnd* Genes	Queried by *ssp* Genes
Gut system	*Escherichia coli* MS 45-1	*Dysgonomonas mossii DSM* 22836
	*Escherichia coli* MS 117-3	*Alistipes indistinctus* YIT 12060
	*Enterobacter cloacae* NCTC 9394	*Prevotella* HGA0225
	*Escherichia coli* SE11	*Bacteroides dorei* 5_1_36/D4
	*Lachnospiraceae bacterium* 6_1_37FAA	*Bacteroides* 1_1_14
	*Desulfovibrio piger* ATCC 29098	*Roseburia inulinivorans* DSM 16841
	*Helicobacter bilis* ATCC 43879	*Roseburia intestinalis* M50/1
	*Paraprevotella xylaniphila* YIT 11841	*Roseburia intestinalis* L1-82
	*Lachnospiraceae bacterium* 2_1_58FAA	*Eubacterium ventriosum* ATCC 27560
	*Clostridium asparagiforme* DSM 15981	*Eubacterium siraeum* V10Sc8a
	*Peptoclostridium difficile* 70-100-2010	*Megamonas funiformis* YIT 11815
	*Bacteroides* 2_1_33B	*Mitsuokella multacida* DSM 20544
	*Bacteroides xylanisolvens* XB1A	*Butyrivibrio fibrisolvens* 16/4
		*Clostridium citroniae* WAL-17108
		*Faecalibacterium prausnitzii* M21/2
		*Fusobacterium necrophorum*
		*funduliforme* 1_1_36S
Oral system	*Neisseria* 020	*Prevotella salivae* F0493
	*Neisseria subflava* NJ9703 N	*Prevotella* F0055
	*Neisseria bacilliformis* ATCC BAA-1200	*Prevotella tannerae* ATCC 51259
	*Eikenella corrodens* ATCC 23834	
	*Lachnospiraceae bacterium* F0431	
	*Selenomonas* CM52	
Urogenital system	*Prevotella amnii* CRIS 21A-A	*Prevotella oralis* ATCC 33269
	*Mycobacterium parascrofulaceum* ATCC BAA-614	*Prevotella denticola* CRIS 18C-A
	*Prevotella bivia* JCVIHMP010	
Skin		*Acinetobacter baumannii* 6014059

**Table 2:** PT-linked dinucleotides in the human fecal DNA.

PT-Linked Dinucleotides	Precursor Ion	Product Ion	z1	z2	z3	1488	1489	1494	1493
d(C_ps_G)	573	152	Y			Y			N
d(C_ps_C)	533	112		Y	Y		Y	Y	
d(G_ps_G)	613	152		Y	Y		Y	Y	
d(C_ps_A)	557	136		Y *	Y *		Y *	Y *	
d(C_ps_T)	548	112	N **	N **	Y	N **	N	Y	
d(A_ps_G)	597	136	Y			Y			
d(T_ps_G)	588	152	Y		Y	Y		Y	
d(G_ps_A)	597	136			Y			Y	
d(G_ps_C)	573	112		N	Y		N	Y	
d(G_ps_T)	588	152	Y	N	Y	Y	N	Y	
d(A_ps_A)	581	136							
d(T_ps_A)	572	136		Y			Y		N
d(A_ps_C)	557	112		Y *	Y *		Y *	Y *	
d(T_ps_C)	548	112		N **	Y		N	Y	
d(A_ps_T)	572	136		Y	Y		Y	Y	N
d(T_ps_T)	563	127							
d(G_ps_A) S_p_	597	136							

Blank means not detected; “Y” means sufficient levels to be quantified; “N” means close to detection limit; “*” means retention time slightly shifted from expected values; “**” means retention time shift.

## Supplementary Materials

The following are available online at https://www.mdpi.com/2218-273X/10/8/1175/s1, Supplementary data and Datasets.
