# Supplementary material for: DNA Phosphorothioate Modifications Are Widely Distributed in the Human Microbiome
Source: Biomolecules. 2020 Aug 12;10(8):1175. doi: 10.3390/biom10081175 (PMC7464106; doi:10.3390/biom10081175)
Supplement: Supplementary file 1 [file biomolecules-10-01175-s001.pdf]

Figure S1. The variation of *dndC*/*sspD* genes**.** Query genes were from *S. enterica serovar* Cerro 87 (CP008925: 3477655...3481641) and *V. cyclitrophicus* FF75 (NZ_ATLT01000001: 2194844...2200061) respectively.

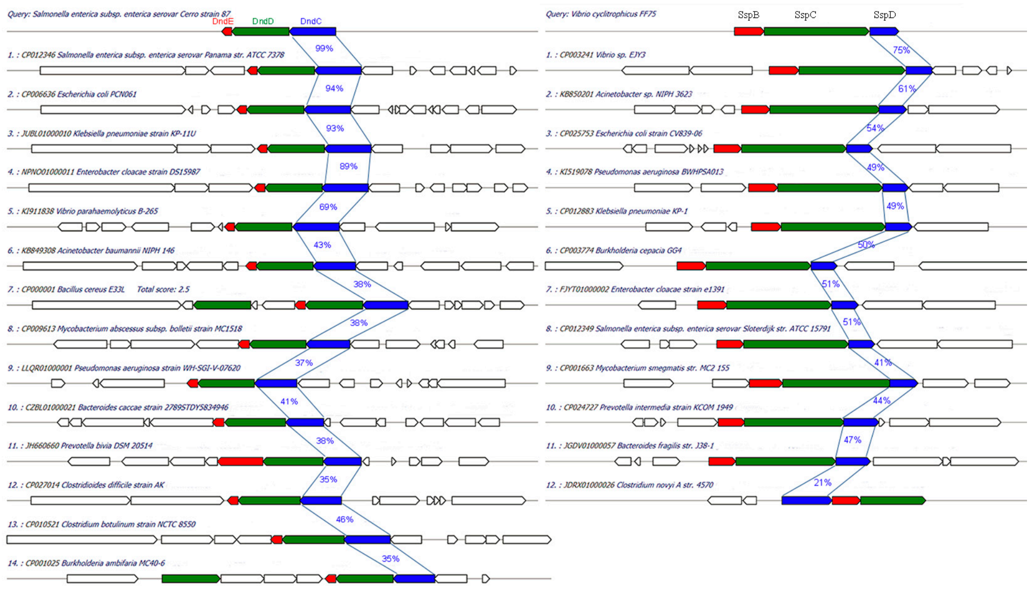

Figure S2. Original data for the qualitation of PT-linked dinucleotides.

S2-1 Prepurification of fecal DNA by high performance liquid chromatography

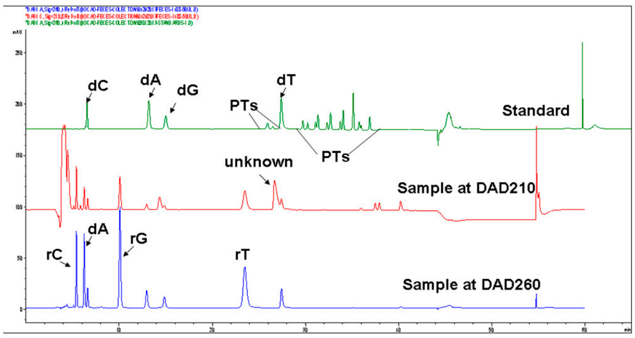

S2-2 The detections of PT-linked dinucleotides by Triple Quadrupole mass spectrometer.

d ($C_{ps}G$) – 16.9 min

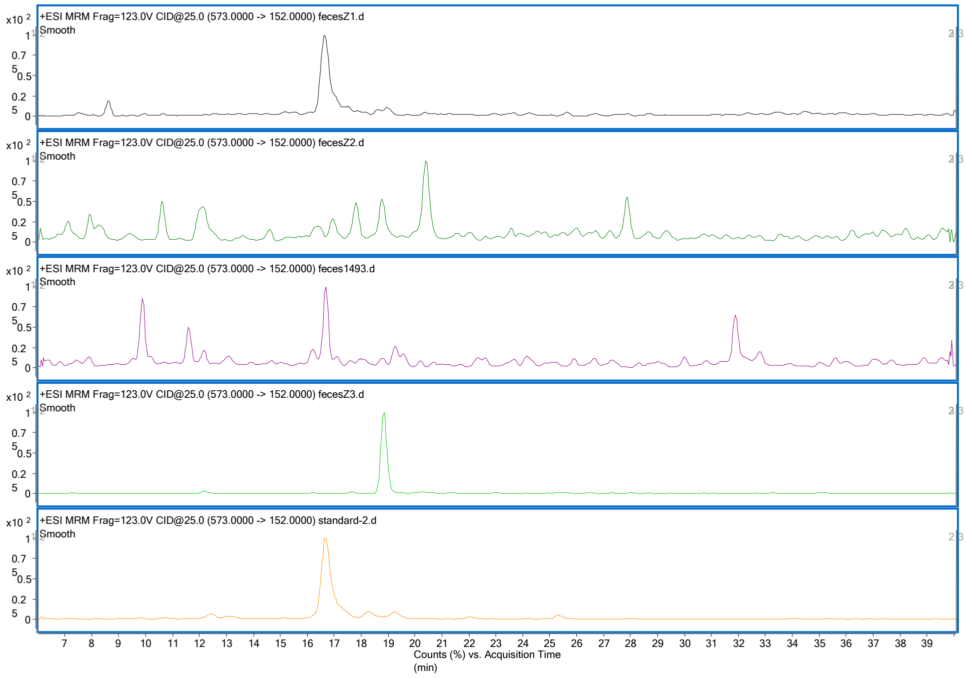

S2-2-1 The detection of $C_{ps}G$ in the fecal DNA (fagmentor voltage: 123 V, precusor ion: 573 m/z, product ion: 152 m/z).

d ($C_{ps}C$) - 11.8 min

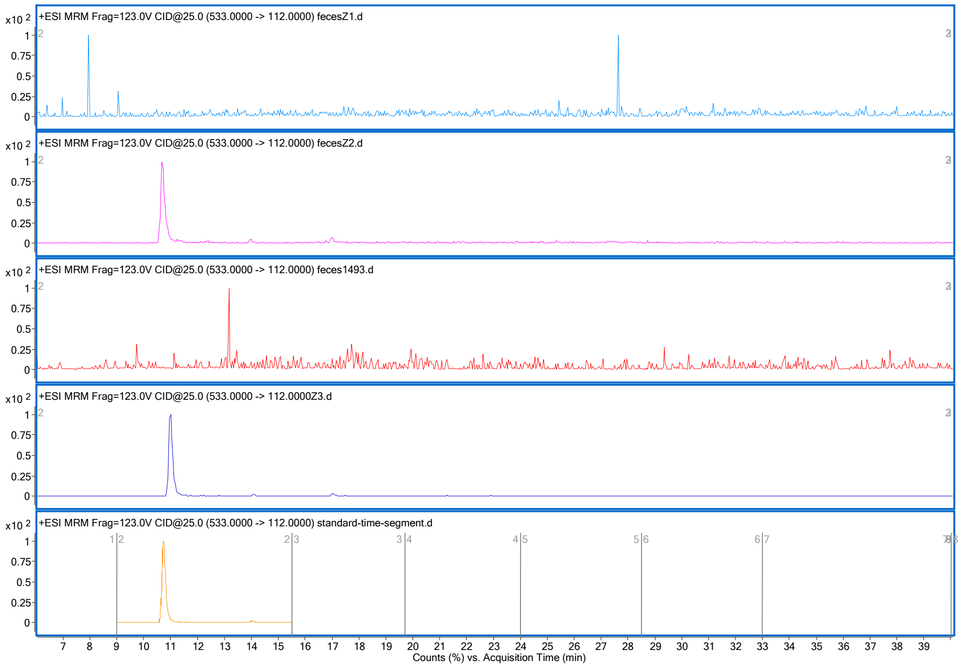

S2-2-2 The detection of $C_{ps}C$ in the fecal DNA (fagmentor voltage: 123 V, precusor ion: 533 m/z, product ion: 112 m/z).

d ($G_{ps}G$) - 19 min

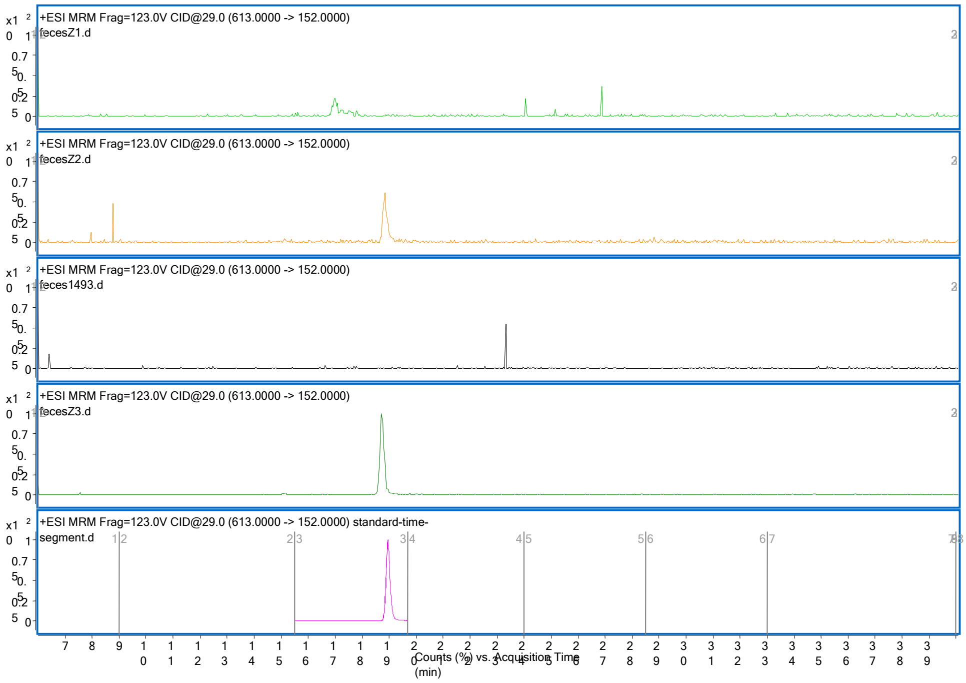

S2-2-3 The detection of $G_{ps}G$ in the fecal DNA (fagmentor voltage: 123 V, precusor ion: 613 m/z, product ion: 152 m/z).

d ($C_{ps}A$) - 14.5 min; d ($A_{ps}C$) - 21 min

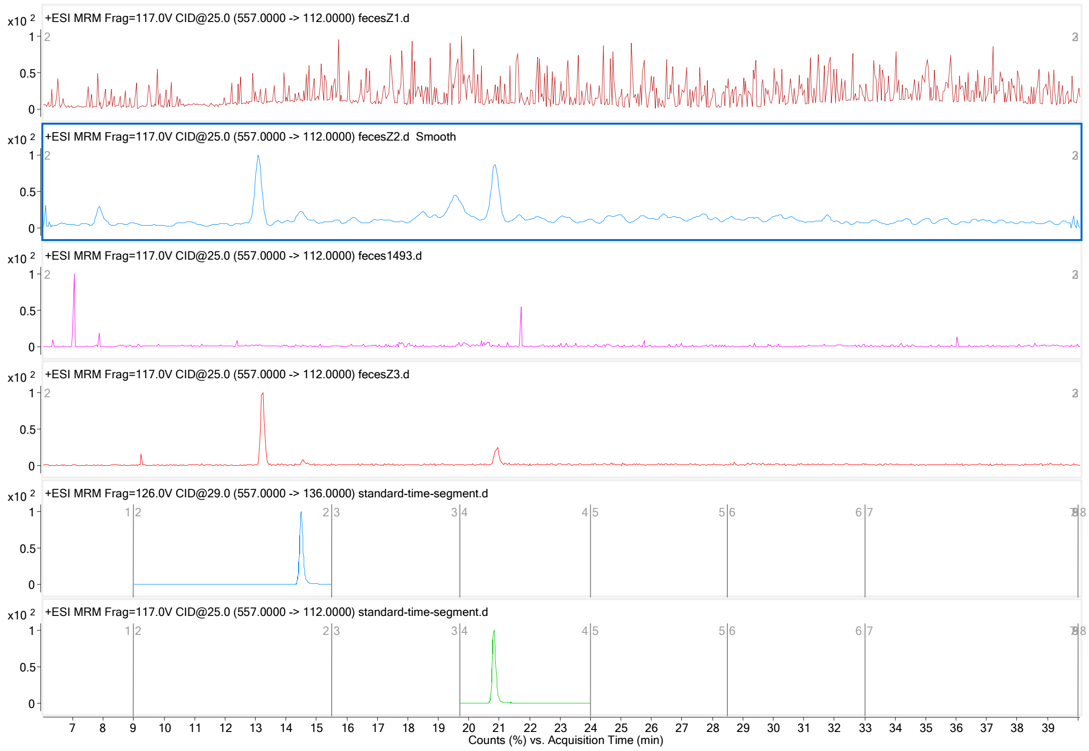

S2-2-4 The detection of $C_{ps}A$ / $A_{ps}C$ in the fecal DNA (fagmentor voltage: 117 V, precusor ion: 557 m/z, product ion: 112 m/z).

d ($C_{ps}T$) - 17.5 min; d ($T_{ps}C$) - 22.5 min

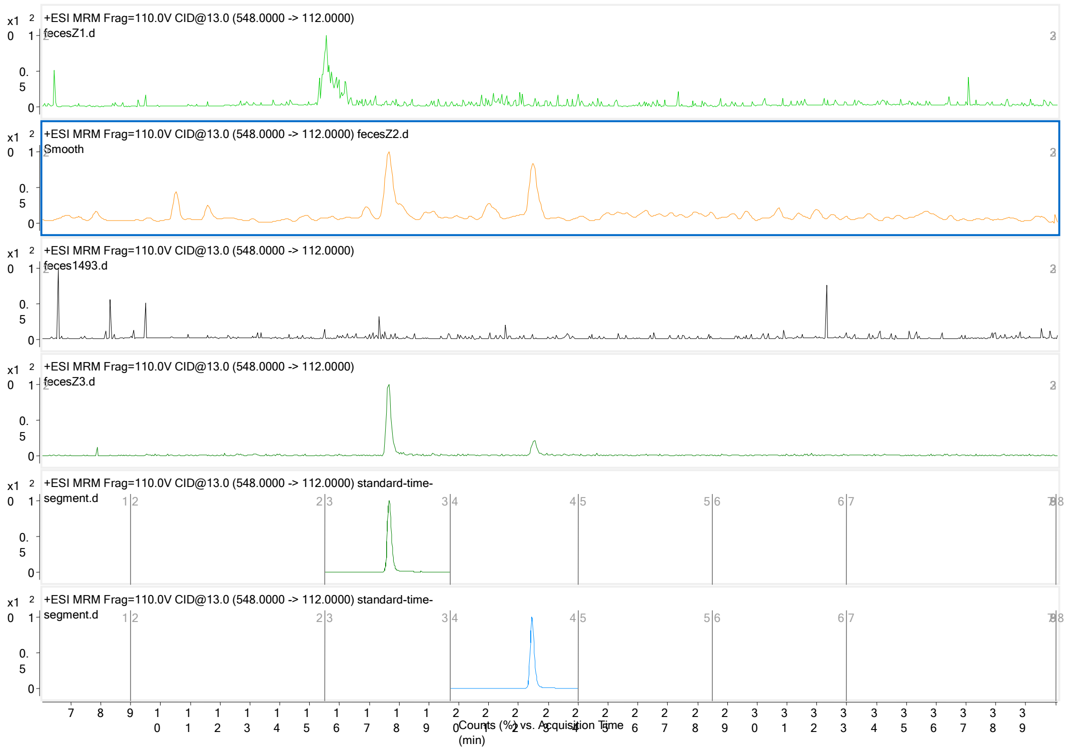

S2-2-5 The detection of $C_{ps}T$ / $T_{ps}C$ in the fecal DNA (fagmentor voltage: 110 V, precusor ion: 548 m/z, product ion: 112 m/z).

d ($A_{ps}G$) - 19 min

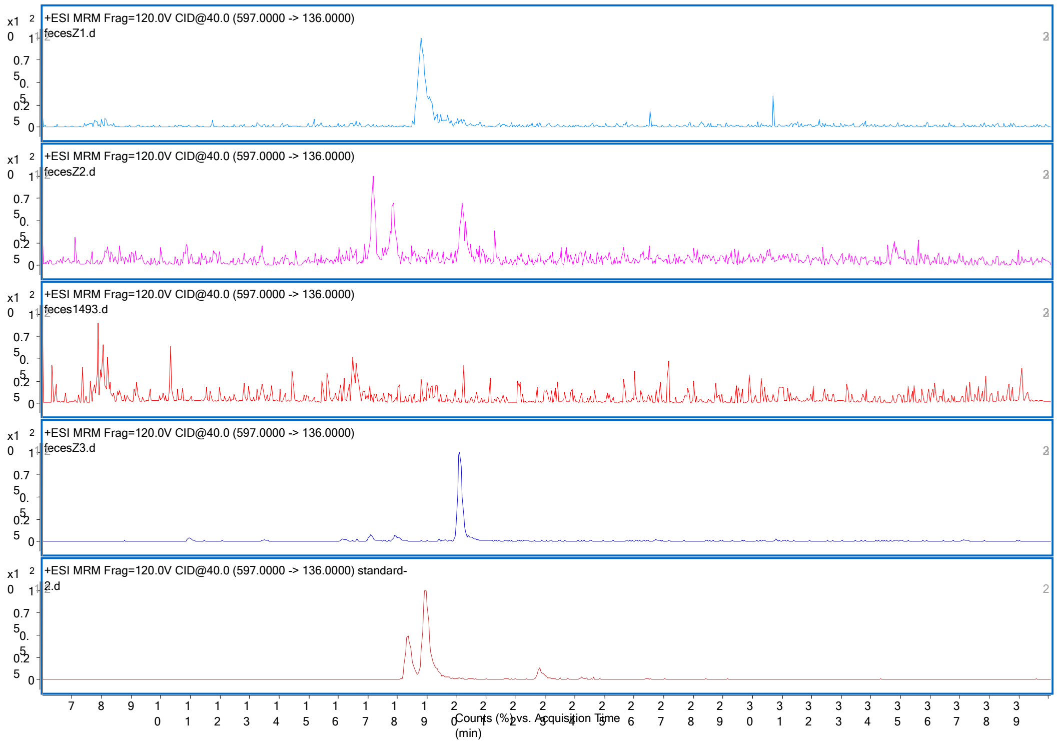

S2-2-6 The detection of $A_{ps}G$ in the fecal DNA (fagmentor voltage: 120 V, precusor ion: 597 m/z, product ion: 136 m/z).

d ($T_{ps}G$) - 25.2 min

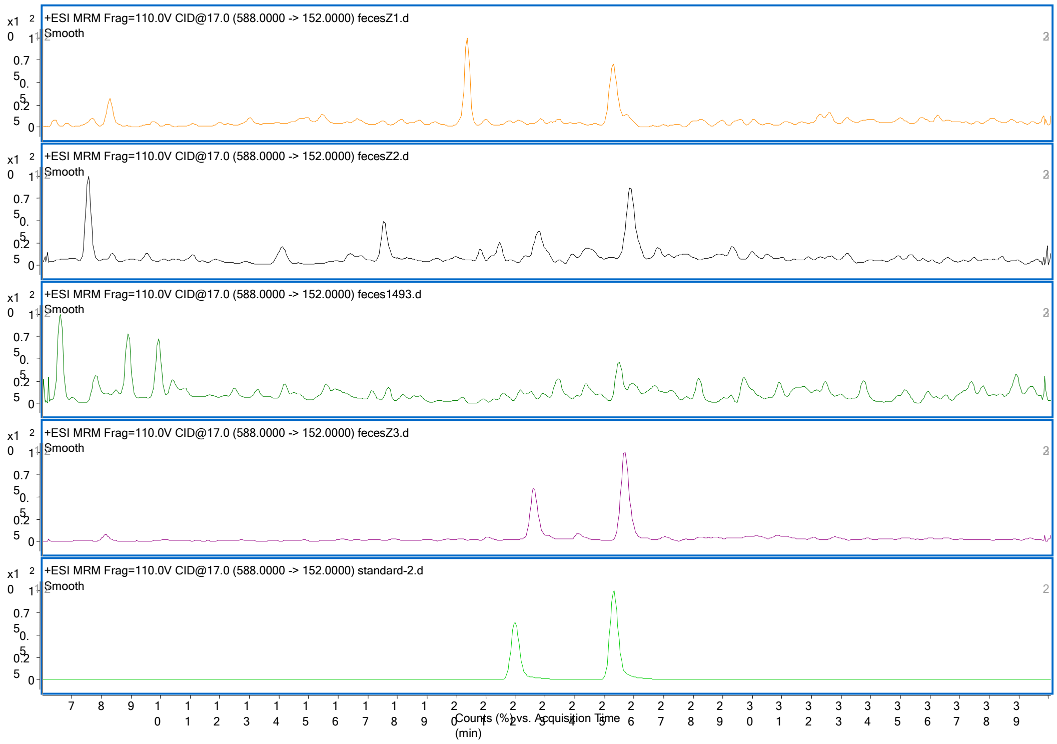

S2-2-7 The detection of $T_{ps}G$ in the fecal DNA (fagmentor voltage: 110 V, precusor ion: 588 m/z, product ion: 152 m/z).

d (G$_{ps}$A) - 20.1 min

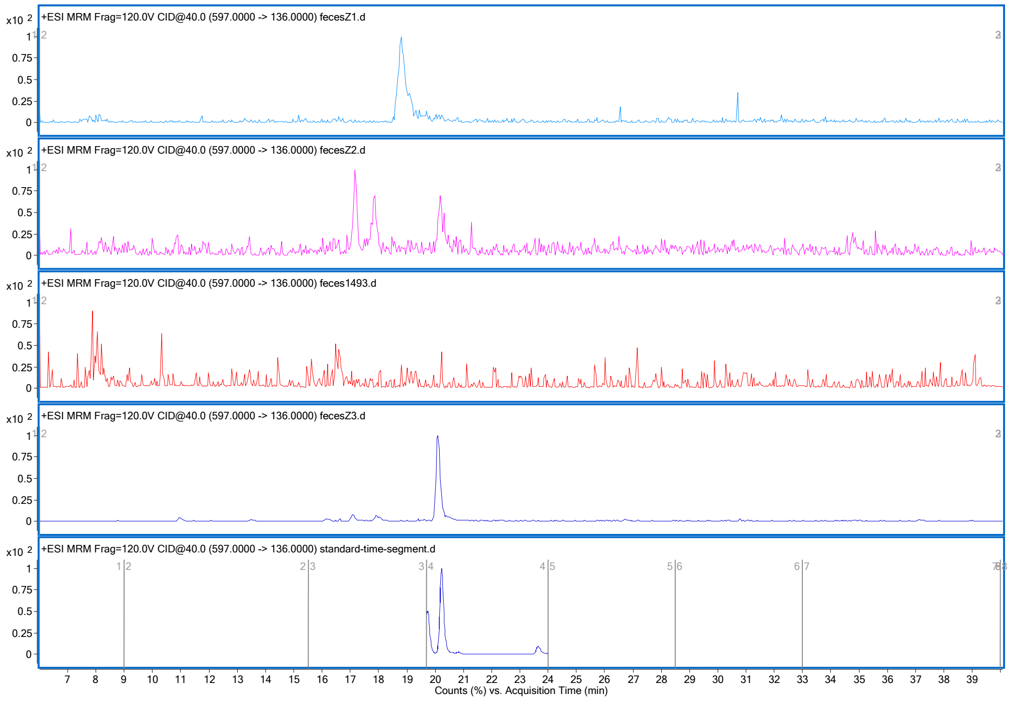

S2-2-8 The detection of G$_{ps}$A in the fecal DNA (fagmentor voltage: 120 V, precusor ion: 597 m/z, product ion: 136 m/z).

d (G$_{ps}$C) - 19 min

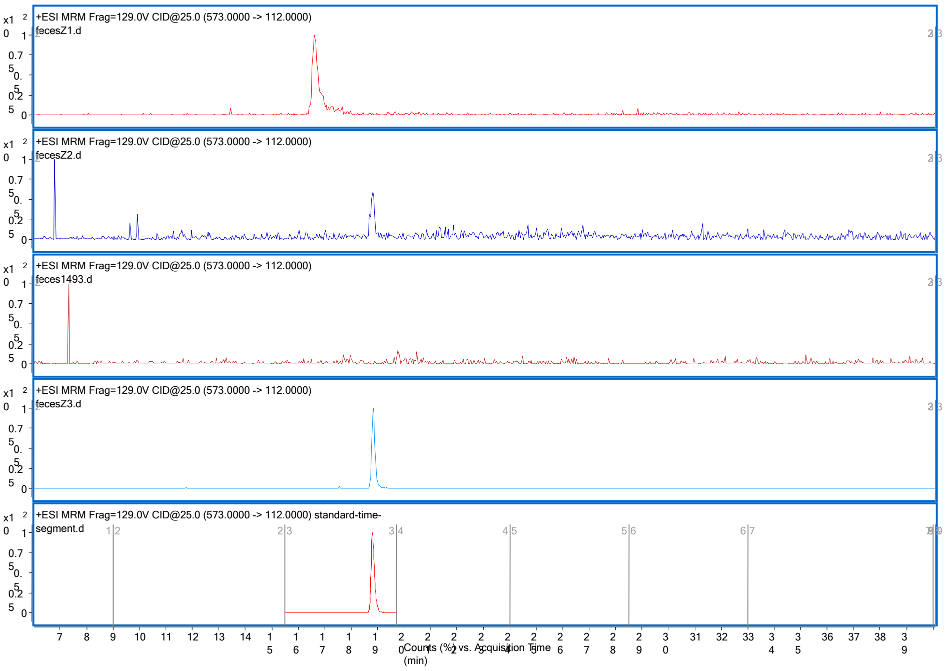

S2-2-9 The detection of G$_{ps}$C in the fecal DNA (fagmentor voltage: 129 V, precusor ion: 573 m/z, product ion: 112 m/z).

d ($G_{ps}T$) - 26 min

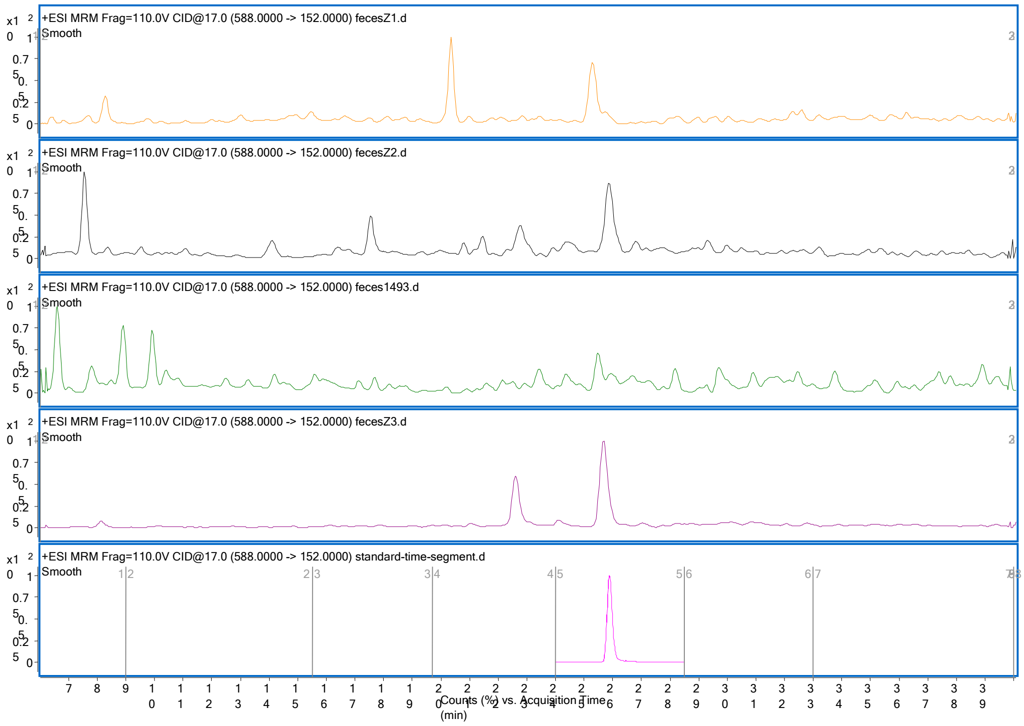

S2-2-10 The detection of $G_{ps}T$ in the fecal DNA (fagmentor voltage: 110 V, precusor ion: 588 m/z, product ion: 152 m/z).

d ($A_{ps}A$) - 21.8 min

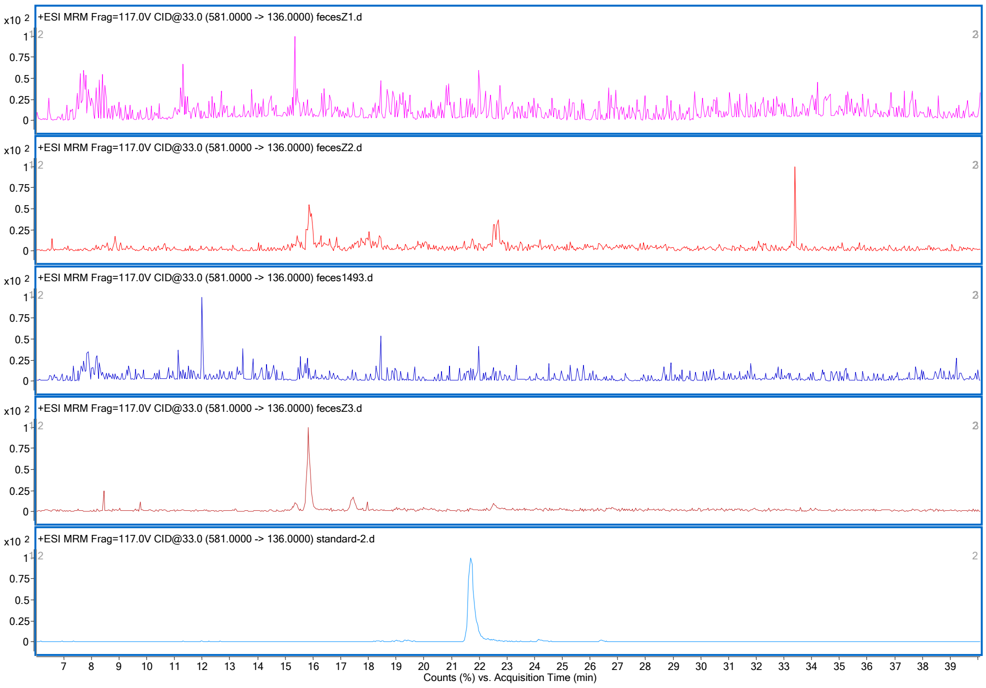

S2-2-11 The detection of $A_{ps}A$ in the fecal DNA (fagmentor voltage: 117 V, precusor ion: 581 m/z, product ion: 136 m/z).

d ($T_{ps}A$) - 24 min; d ($A_{ps}T$) - 26.5 min

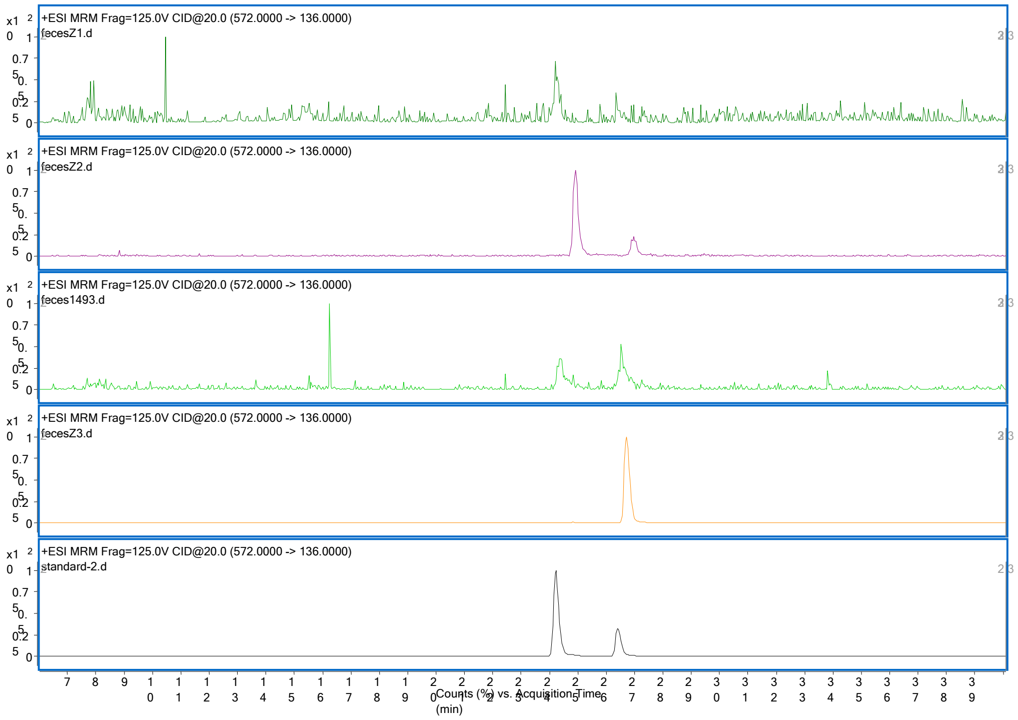

S2-2-12 The detection of $T_{ps}A$ / $A_{ps}T$ in the fecal DNA (fagmentor voltage: 125 V, precusor ion: 572 m/z, product ion: 136 m/z).

d ($C_{ps}G$) - 16.8 min

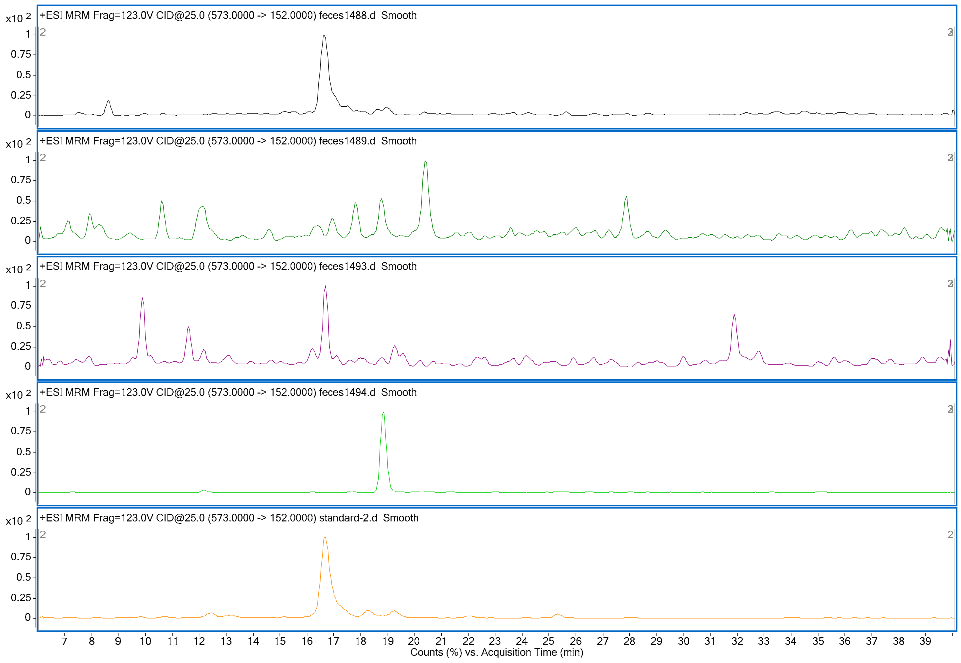

S2-2-13 The detection of $C_{ps}G$ in the fecal DNA (fagmentor voltage: 123 V, precusor ion: 573 m/z, product ion: 152 m/z).

d ($C_{ps}C$) - 10.9 min

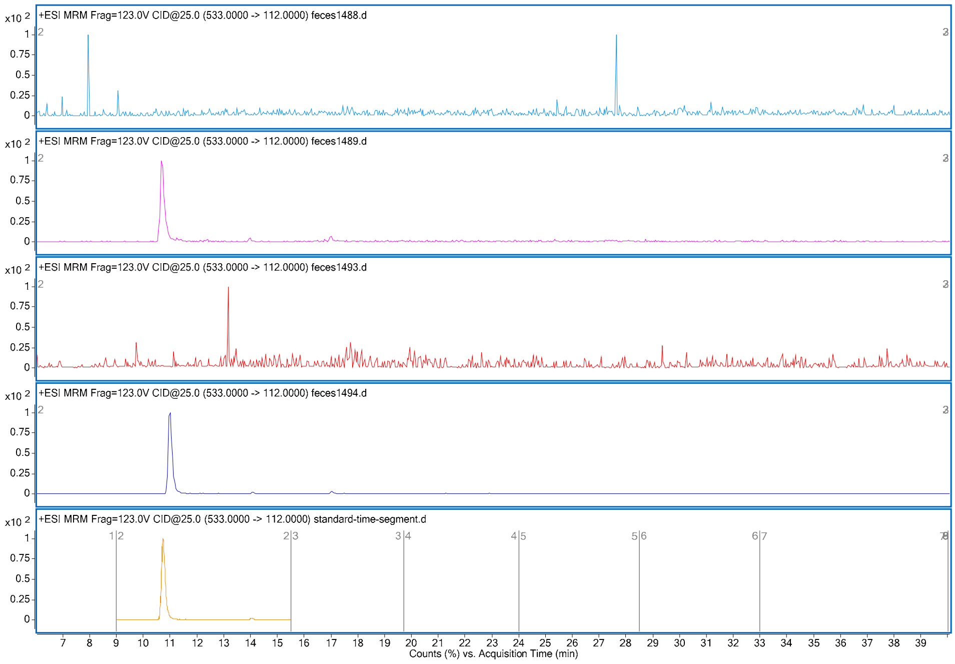

S2-2-14 The detection of $C_{ps}C$ in the fecal DNA (fagmentor voltage: 123 V, precusor ion: 533 m/z, product ion: 112 m/z).

d ($G_{ps}G$) -19 min

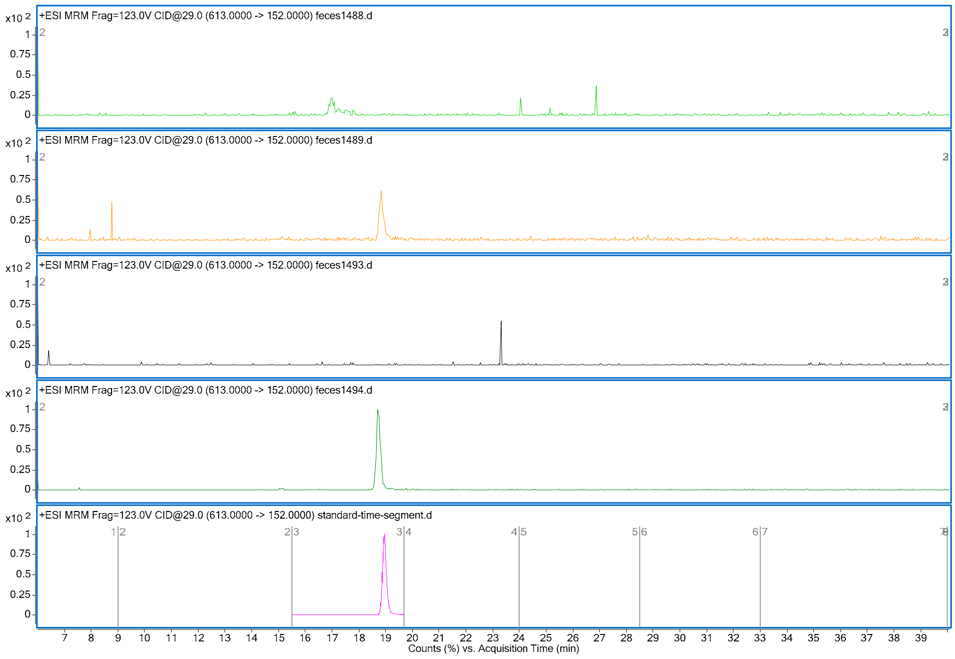

S2-2-15 The detection of $G_{ps}G$ in the fecal DNA (fagmentor voltage: 123 V, precusor ion: 613 m/z, product ion: 152 m/z).

d ($C_{ps}A$) -14.5 min; d ($A_{ps}C$) - 21 min

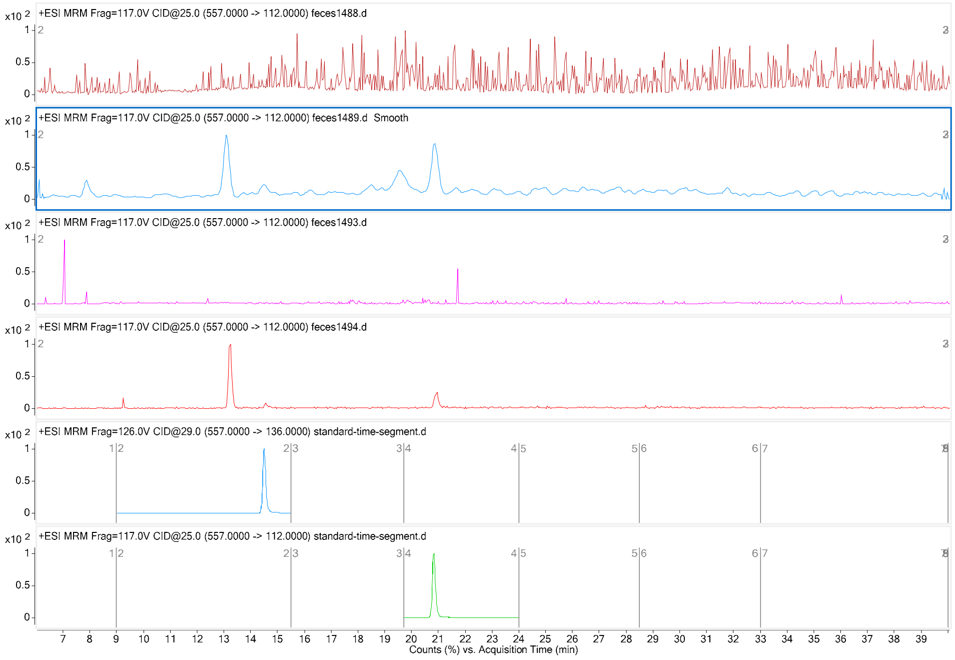

S2-2-16 The detection of $C_{ps}A$ / $A_{ps}C$ in the fecal DNA (fagmentor voltage: 117 V, precusor ion: 557 m/z, product ion: 112 m/z).

d ($C_{ps}T$) - 17.5 min; d ($T_{ps}C$) - 22.5 min

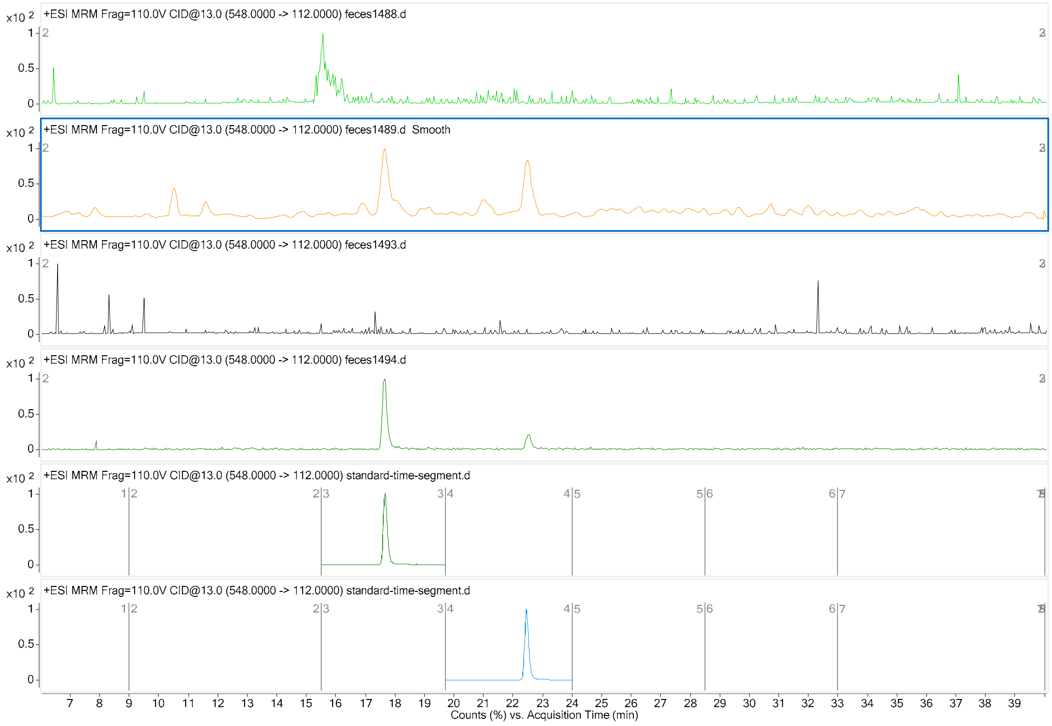

S2-2-17 The detection of $C_{ps}T$ / $T_{ps}C$ in the fecal DNA (fagmentor voltage: 110 V, precusor ion: 548 m/z, product ion: 112 m/z).

d ($A_{ps}G$) - 19 min

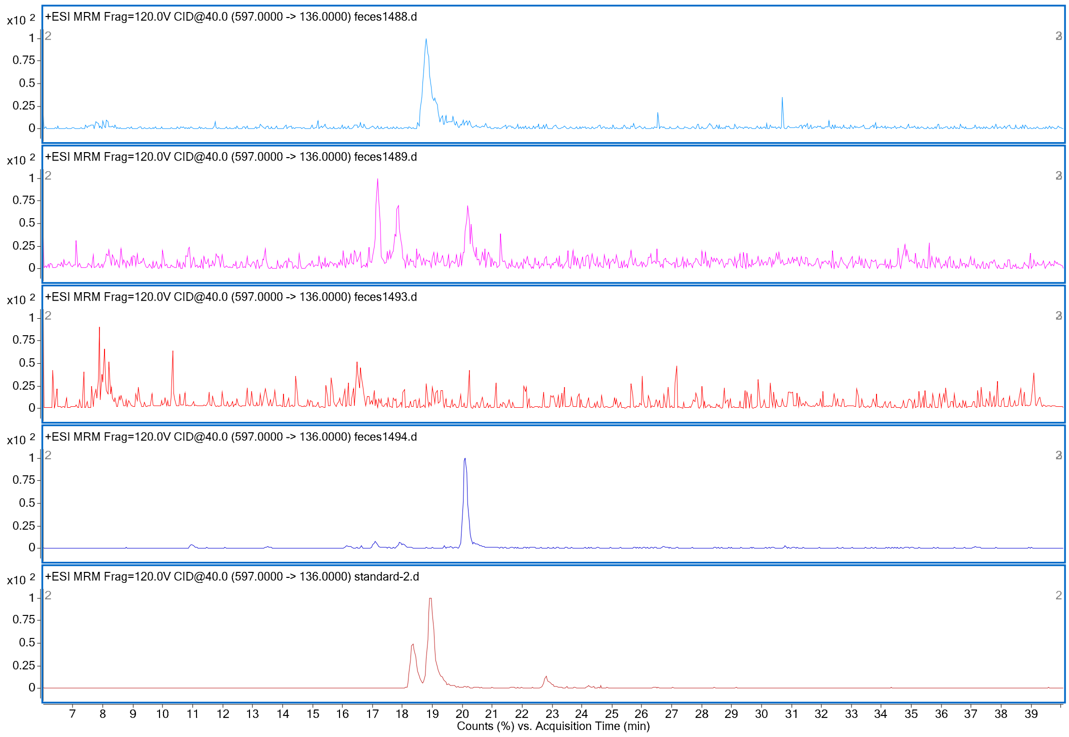

S2-2-18 The detection of $A_{ps}G$ in the fecal DNA (fagmentor voltage: 120 V, precusor ion: 597 m/z, product ion: 136 m/z).

d ($T_{ps}G$) - 25.3 min

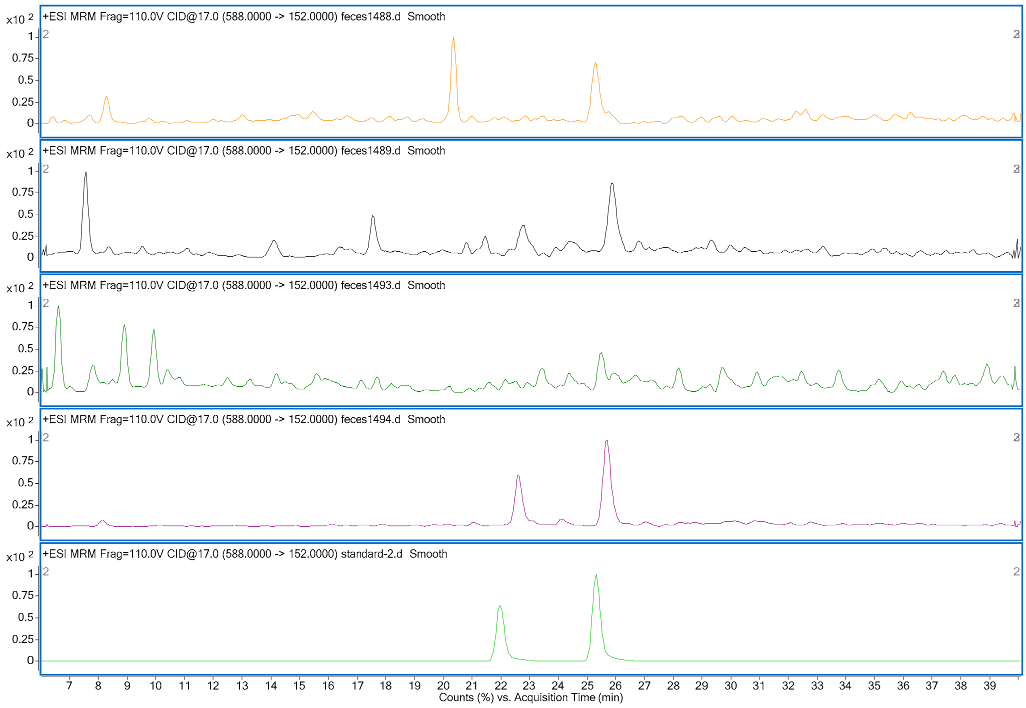

S2-2-19 The detection of $T_{ps}G$ in the fecal DNA (fagmentor voltage: 110 V, precusor ion: 588 m/z, product ion: 152 m/z).

d ($G_{ps}A$) - 21.1 min

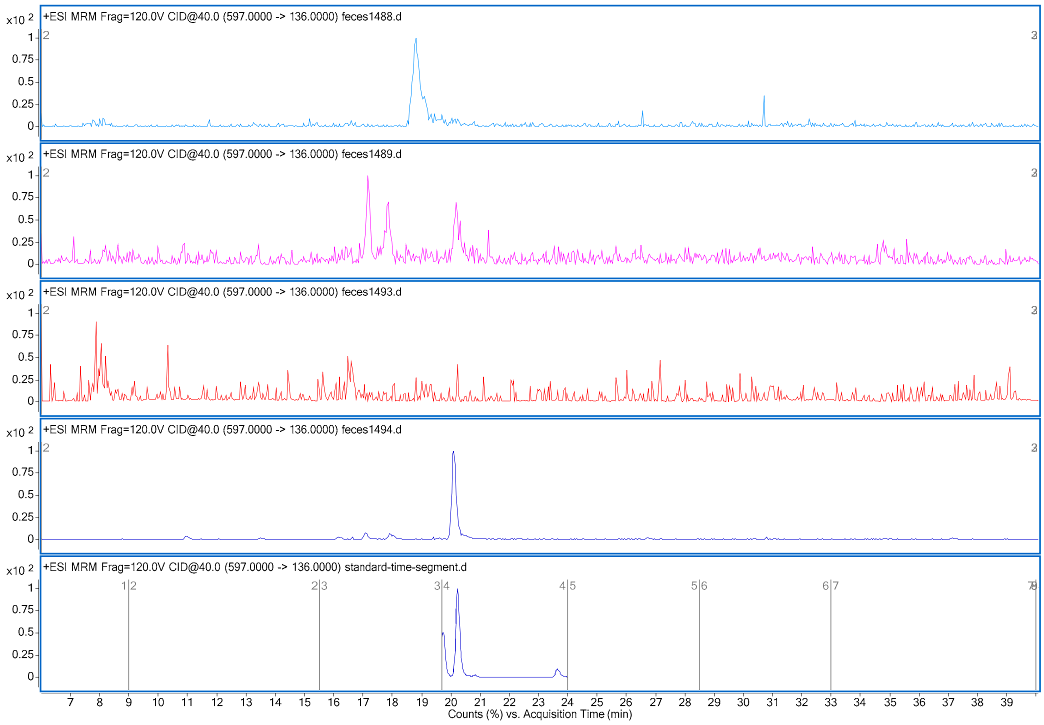

S2-2-20 The detection of $G_{ps}A$ in the fecal DNA (fagmentor voltage: 120 V, precusor ion: 597 m/z, product ion: 136 m/z).

d ($G_{ps}C$) - 19 min

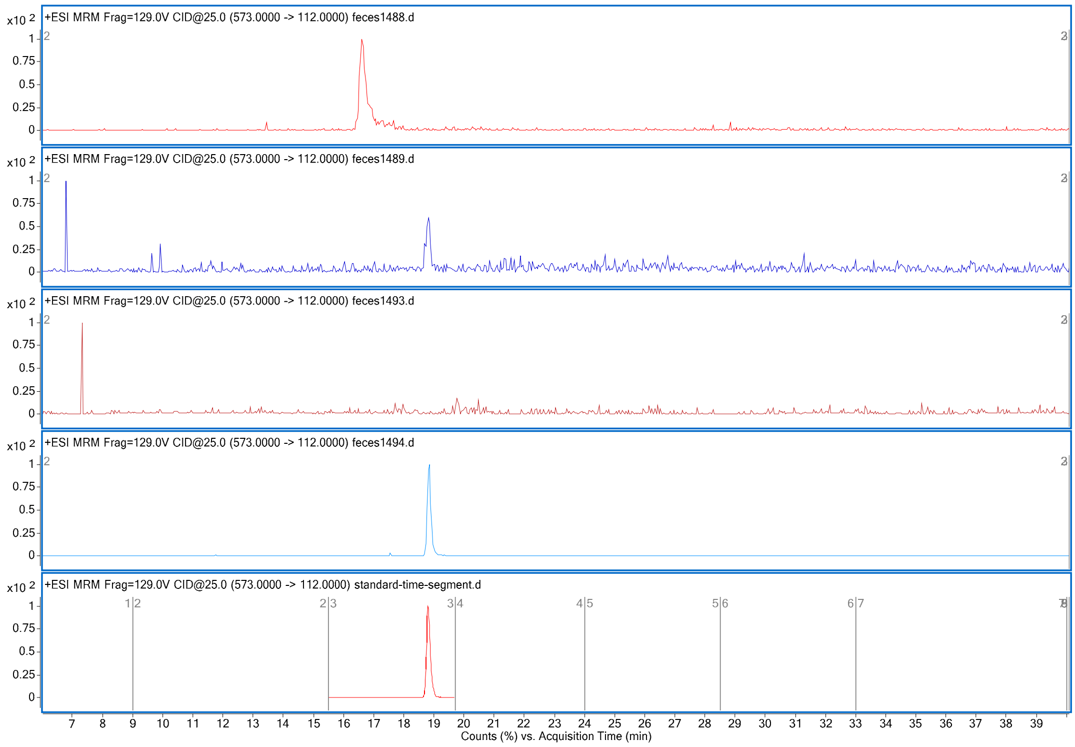

S2-2-21 The detection of $G_{ps}C$ in the fecal DNA (fagmentor voltage: 129 V, precusor ion: 573 m/z, product ion: 112 m/z).

d ($G_{ps}T$) - 26 min

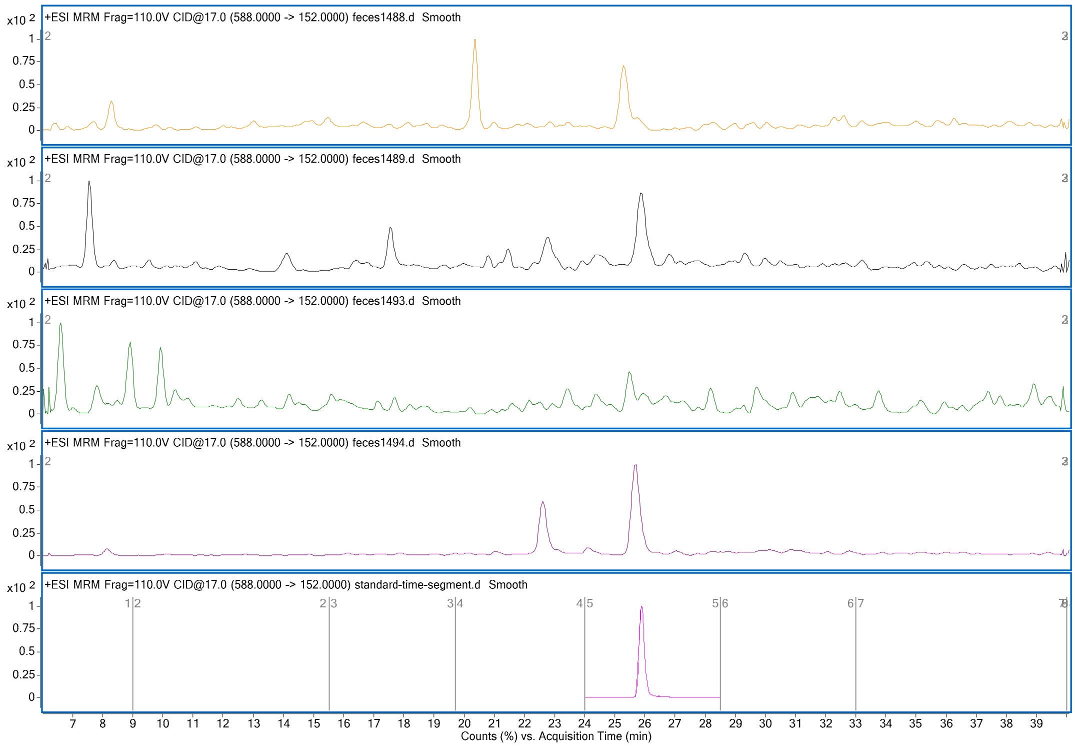

S2-2-22 The detection of $G_{ps}T$ in the fecal DNA (fagmentor voltage: 110 V, precusor ion: 588 m/z, product ion: 152 m/z).

d ($A_{ps}A$) - 21.9 min

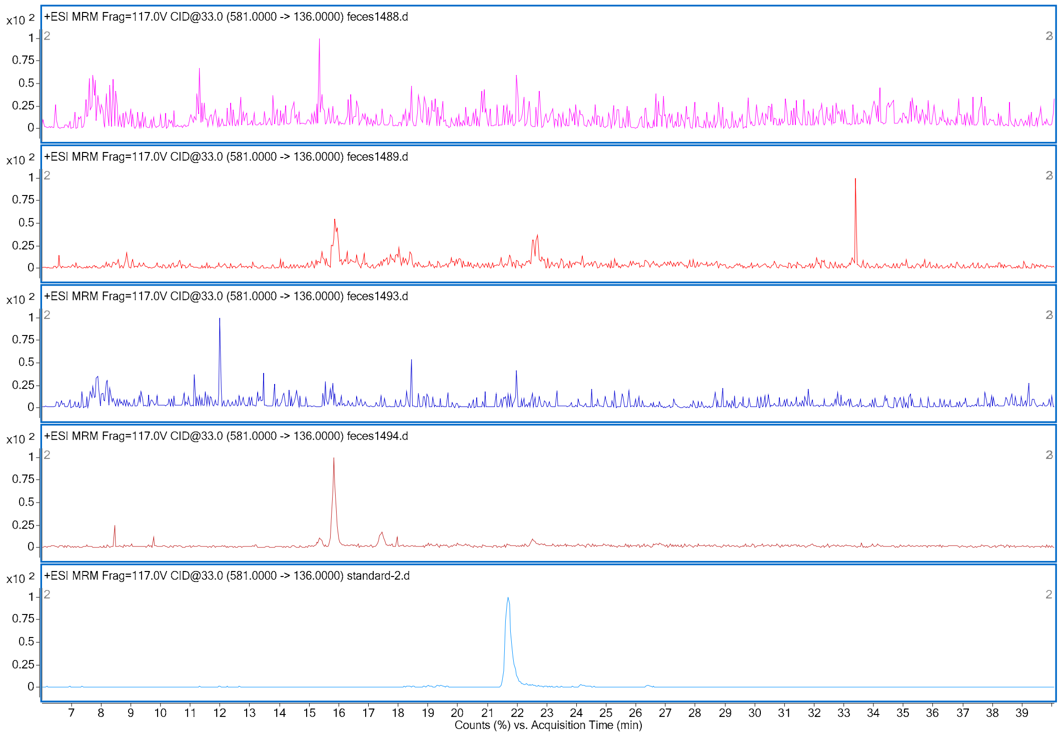

S2-2-23 The detection of $A_{ps}A$ in the fecal DNA (fagmentor voltage: 117 V, precusor ion: 581 m/z, product ion: 136 m/z).

d ($T_{ps}A$) - 24 min; d($A_{ps}T$) - 26.5 min

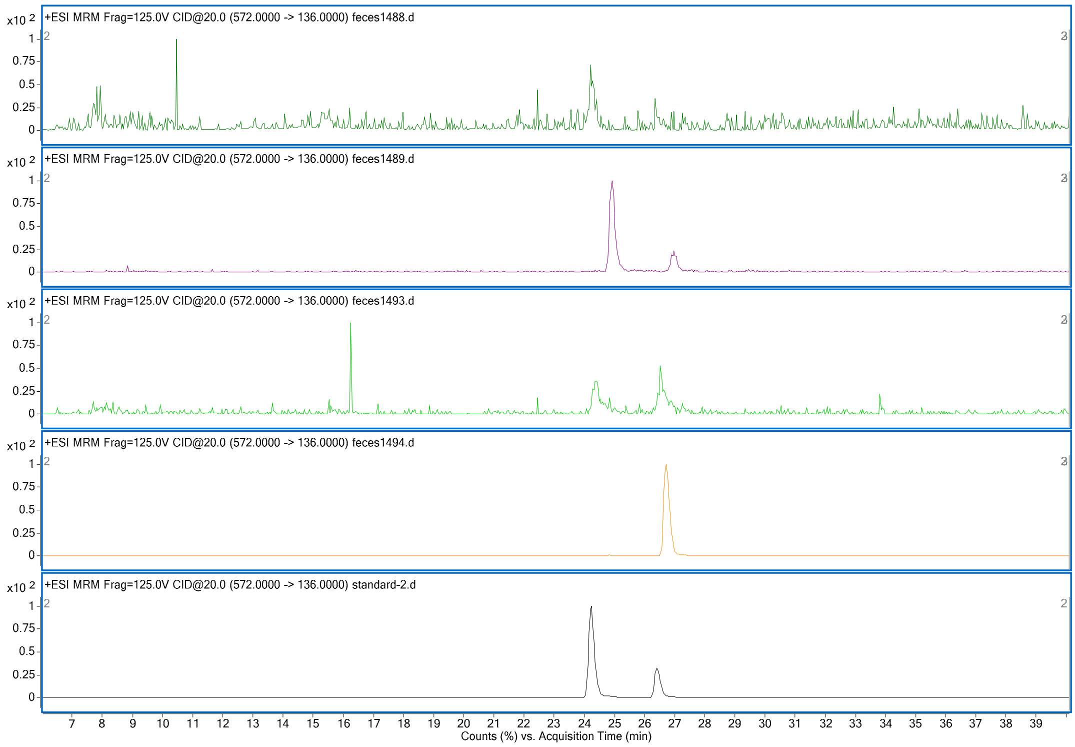

S2-2-24 The detection of $T_{ps}A$/ $A_{ps}T$ in the fecal DNA (fagmentor voltage: 125 V, precusor ion: 572 m/z, product ion: 136 m/z).

d ($A_{ps}C$) - 21 min

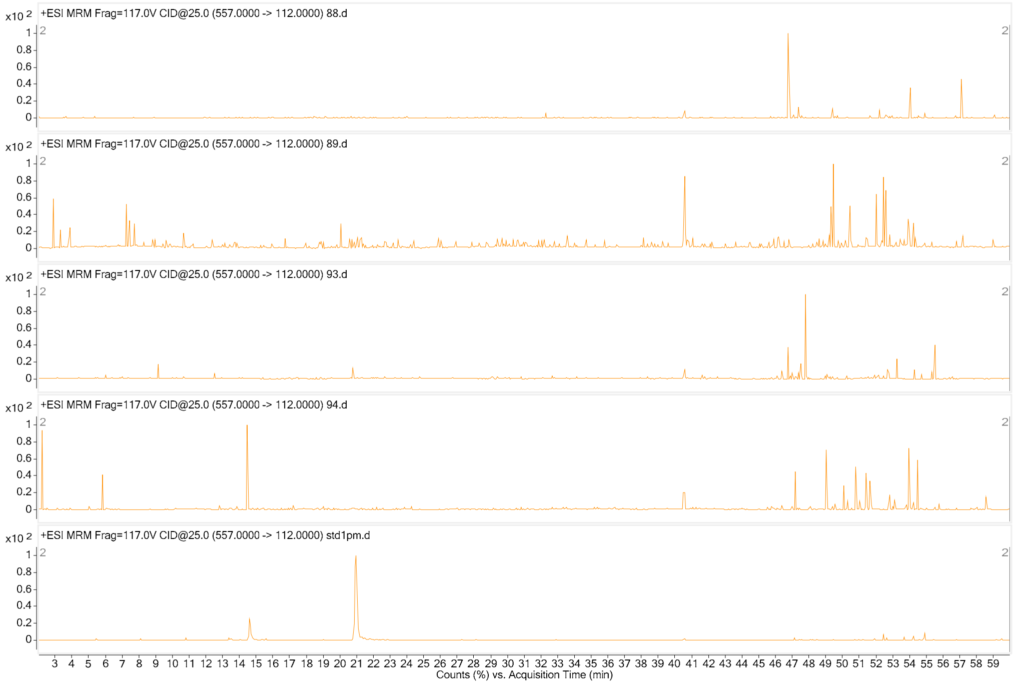

S2-2-25 The detection of $A_{ps}C$ in the fecal DNA (fagmentor voltage: 117 V, precusor ion: 557 m/z, product ion: 112 m/z).

d ($T_{ps}C$) - 18 min

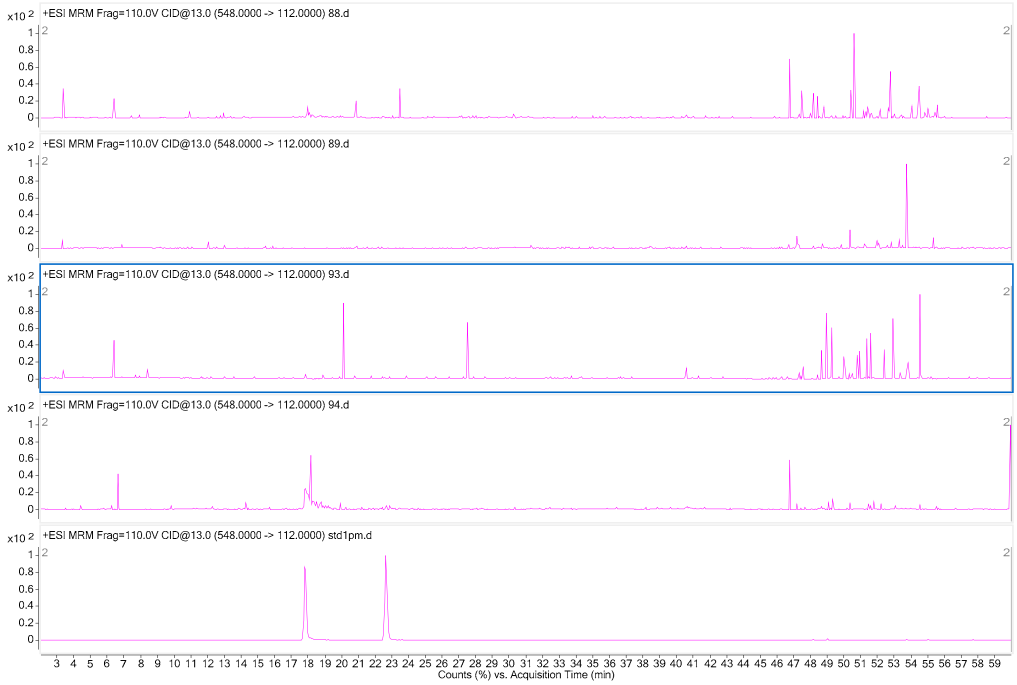

S2-2-26 The detection of $T_{ps}C$ in the fecal DNA (fagmentor voltage: 110 V, precusor ion: 548m/z, product ion: 112 m/z).

d ($T_{ps}T$) - 30.4 min

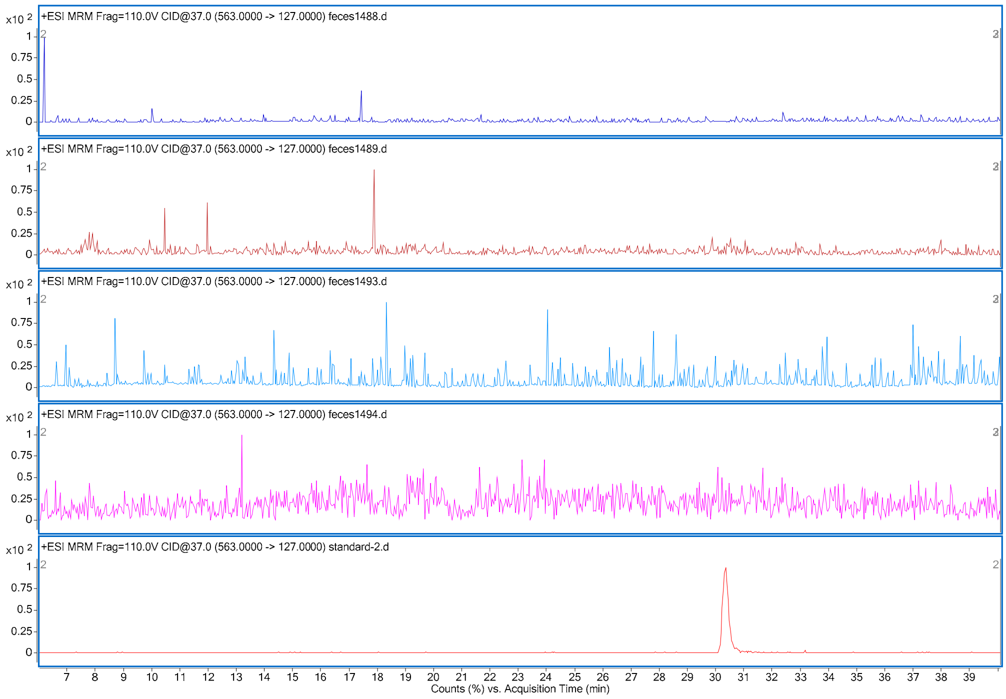

S2-2-27 The detection of $T_{ps}T$ in the fecal DNA (fagmentor voltage: 110 V, precusor ion: 563 m/z, product ion: 127 m/z).
